# Structural and interagency influences on mandated health assessments for children in out-of-home care in Sweden: a qualitative study of child protection services workers

**DOI:** 10.1136/bmjpo-2026-005108

**Published:** 2026-09-25

**Authors:** Godfried Van Agthoven, Gabriel Otterman, Rajna Knez, Kathi Makoroff, Laura Korhonen

**Affiliations:** 1 Swedish National Competence Centre on Violence Against Children and Department of Biomedical and Clinical Sciences, Linköping University, Linköping, Sweden; 2 Department of Pediatrics, Skaraborg Hospital Skövde, Skövde, Sweden; 3 School of Health Sciences, University of Skövde, Skövde, Sweden; 4 Mayerson Center for Safe and Healthy Children, Cincinnati Children’s Hospital Medical Center, Cincinnati, Ohio, USA; 5 Department of Pediatrics, University of Cincinnati College of Medicine, Cincinnati, Ohio, USA; 6 Department of Child and Adolescent Psychiatry, Linköping University, Linkoping, Sweden

**Keywords:** Qualitative research, Child Health, Health Policy, Health services research, Social work

## Abstract

**Background:**

Children in out-of-home care (OOHC) have significantly poorer physical, mental and dental health than their peers. In Sweden, child protection services (CPS) are required to initiate health assessments (HAs) when a child is placed in OOHC. Despite this statutory requirement, fewer than half of eligible children undergo an HA. Factors influencing implementation remain poorly understood.

**Methods:**

This qualitative study explored CPS caseworkers’ experiences of initiating and coordinating HAs for children placed in OOHC in Sweden. Five semi-structured focus group discussions were conducted with 18 CPS caseworkers. Participants were recruited nationwide through purposive snowball sampling. Data were analysed using reflexive thematic analysis, applying a decision-making ecology framework to interpret individual, organisational and interagency influences.

**Results:**

Four interrelated themes captured factors influencing implementation across the HA pathway: (1) Ideally, we do it for the child (initiation); (2) Negotiation and uncertainty (scheduling); (3) What we request is not always what we receive (conduct); and (4) Variable reporting and the challenge of closing the loop (reporting and follow-up). Across all phases, CPS caseworkers assumed a de facto coordinating role to compensate for fragmented interagency structures.

**Conclusions:**

From the perspective of CPS caseworkers, organisational and interagency factors influenced HAs across the assessment pathway. Fragmented routines and unclear responsibilities may reduce their usefulness for care planning and follow-up. Strengthening shared ownership and improving assessment, reporting and follow-up processes may support implementation. HAs may be most useful as part of a broader pathway of assessment, referral, care planning and follow-up.

**WHAT IS ALREADY KNOWN ON THIS TOPIC**

- Children in out-of-home care have substantially poorer physical, mental and dental health than their peers. Although health assessments (HAs) are legally mandated in Sweden, uptake remains low and factors influencing implementation are not well understood.

**WHAT THIS STUDY ADDS**

- This qualitative study suggests that implementation of HAs is influenced by organisational and interagency factors across the assessment pathway. Child protection services (CPS) caseworkers described assuming a de facto coordinating role to manage fragmented routines, unclear responsibilities and variation in assessment, reporting and follow-up practices.

**HOW THIS STUDY MIGHT AFFECT RESEARCH, PRACTICE OR POLICY**

- The findings highlight opportunities for stronger shared ownership between CPS and healthcare, clearer allocation of responsibilities and more structured pathways for assessment, referral and follow-up. HAs may be useful when embedded within a coordinated pathway rather than conceptualised as isolated examinations.

## Introduction

 Children in out-of-home care (OOHC) experience markedly poorer physical, mental and dental health than their peers, often following exposure to neglect, abuse or family adversity such as substance misuse or criminality.^1–3^ Although OOHC aims to provide safer living conditions, children in care show consistently poorer outcomes than children raised in family settings, including lower academic achievement, higher substance use and increased health problems.^4–7^

In Sweden, children in OOHC are less likely to access preventive healthcare and more likely to have unmet health needs.^8 9^ Register-based studies have demonstrated that children in OOHC are at increased risk of psychiatric disorders, psychotropic medication use, suicide and early exit from the labour market due to mental illness.^10–12^ Recent evidence further links OOHC with increased adult mortality, cardiovascular disease and suicide attempts.^13 14^

To address these health disparities, systematic health assessments (HAs) for children in OOHC have been introduced in several countries, including the UK, USA and Australia.^5 15^ In Sweden, the 2017 Act on Health Examinations of Children and Young People in Out-of-Home Care (SFS 2017:209, Swedish Code of Statutes) mandates that child protection services (CPS) initiate health examinations unless a recent assessment already has been conducted (see online supplemental file 1). National guidelines specify that assessments should include physical, mental and oral health.^16^

Despite this legal mandate, national evaluations by the National Board of Health and Welfare and the Swedish Association of Local Authorities and Regions consistently show low coverage, with fewer than half of eligible children receiving a HA.^16–19^ Identified barriers include administrative burden (eg, consent collection, waiting times), limited digital support (eg, lack of information technology support and digitalised templates) and organisational fragmentation, but existing evaluations provide limited explanatory insight on the mechanisms on how the barriers interact in routine practice.

Although CPS are solely responsible for initiating HAs, no studies have examined implementation from the perspective of CPS caseworkers. Research on interagency collaboration has primarily focused on coordination between CPS and mental health services^20–23^ and highlights challenges related to unclear shared governance and divergent understanding of ‘health’.^23^ Understanding how caseworkers navigate the HA mandate is therefore essential to explain why implementation breaks down in routine practice, particularly in the absence of established organisational models.^24^ Implementation depends on both individual decision-making and on organisational and interagency contexts. The Decision-Making Ecology framework conceptualises these influences across individual, organisational and system levels, providing a conceptual lens for examining how HA are initiated and coordinated in routine practice.^25^

The aim of this study was to explore child protection services caseworkers’ experiences of the initiation, scheduling, conduct, and reporting and follow-up of legally mandated HAs for children in out-of-home care, and to identify structural and interagency factors influencing implementation across the assessment pathway.

## Methods

### Study design

This study used a qualitative design based on semi-structured focus group discussions to explore CPS caseworkers’ experiences of initiating and coordinating HAs for children placed in OOHC.

### Participants and recruitment

CPS caseworkers in Sweden with experience of OOHC placements were recruited using purposive snowball sampling to achieve variation in geographical location and professional experience. Recruitment took place between November 2022 and May 2023 through professional networks, events, National Centre Barnafrid’s website and contact with social services managers. Eligibility required current or recent involvement in OOHC placement decisions. 18 out of 34 eligible CPS caseworkers participated.

### Data collection

A semi-structured interview guide was developed and piloted with three CPS caseworkers prior to data collection (see online supplemental file 2). Five focus group (FG) discussions were conducted by videoconference between November 2022 and June 2023 until meaning-saturation was achieved. Each lasted 90–105 min and was moderated by the male first author, who has prior experience in qualitative interviewing and professional collaboration with child welfare services. At least one additional author was present as a silent observer supporting discussion and reflexive note-taking. No follow-up interviews or participants feedback were obtained.

### Data analysis

Audio recordings were transcribed verbatim and pseudonymised. Data were analysed using reflexive thematic analysis as described by Braun and Clarke, supported by NVivo software.^26 27^ Initial inductive coding by GVA, RK and LK was followed by a second analytic phase (GVA and LK) using the decision-making ecology framework^25^ as a sensitising lens to interpret how individual, organisational and interagency factors influenced implementation across initiation, scheduling, conduct, reporting and follow-up of HAs. Throughout analysis, the research team engaged in reflexive discussions regarding how their professional experiences and assumptions might influence coding, interpretation and theme development.

### Patient and public involvement

Patients and/or the public were not involved in the design or conduct or reporting or dissemination plans of this research.

## Results

### Participants

18 CPS caseworkers participated in the study. Two participants were men (11.1%), corresponding to the proportion of male CPS caseworkers in Sweden. Participants were geographically diverse, representing rural areas (38.9%), urban areas (38.9%) and metropolitan areas (22.2%). Professional experience varied: two participants had less than 5 years of experience, while five had more than 10 years of experience in child protection services.

### Overview of themes across the health assessment pathway

Four interrelated themes matching the phases of the HA pathway captured factors influencing implementation of HA across the assessment pathway: (1) Ideally, we do it for the child (initiation); (2) Negotiation and uncertainty (scheduling); (3) What we request is not always what we receive (conduct); and (4) Variable reporting and the challenge of closing the loop (reporting and follow-up).

Together, these themes describe how HA implementation was shaped by individual, organisational and interagency factors across all phases of the process. Across themes, CPS caseworkers described a coordinating role in managing fragmented routines and unclear responsibilities between CPS and healthcare systems. Table 1 summarises facilitating and challenging factors influencing implementation across the HA pathway.

**Table 1:** Phases, themes, facilitating factors and challenging factors in the health assessment pathway

Phase of health assessment	Theme	Facilitating factors	Challenging factors
Initiation	Ideally, we do it for the child	Legal mandate “Children have the right to good health” FG2R6 Positive prior experiences “A very good doctor…conducts a very good examination” FG2R6 Awareness of elevated needs “Children in care have poorer health” FG5R16	High workload and competing tasks “A lot to do…ends up falling between the cracks” FG5R17 Staff turnover and inexperience “Newly qualified…has no idea” FG2R5 Preconceptions of low utility “A lot of work for nothing” FG5R15
Scheduling	Negotiation and uncertainty	Clear routines and templates “…a well-defined template…” FG5R16 Accountability “…everybody really takes responsibility and knows who is responsible …” FG1R3	Regional variation and unclear contacts “…it varies greatly who you are supposed to contact…” FG3R10 Long waiting times “…waited long…an appointment after nine months…” FG5R14
Conduct	What we request is not always what we receive	Prepared and experienced physicians “A small group of doctors…provide a good assessment” FG1R3 Child-centred and trauma-informed care “Creates a sense of coherence for the child” FG3R10	Limited time “…I don’t think it is reasonable to obtain much information given the limited time…” FG3R9 Limited trauma/violence competence “…violence was not addressed during the examination…” FG2R7
Reporting and follow-up	Variable reporting and the challenge of closing the loop	Structured, actionable reports “Several pages…with bullet points and action items…” FG2R7 Feedback supporting understanding “…it was a teenager. She also thought it was very good. Because she received a lot of information about things her mother had not told her…” FG3R6	Vague or contradictory documentation “…it is hard to understand not having a medical background…” FG5R15 Responsibility drift “…it often ends up being me who has to push it forward…” FG5R14

FG, focus group; R, respondent.

### Initiation of health assessments

#### Theme 1: ideally, we do it for the child

Participants consistently described HA as a rights-based and legally mandated obligation, emphasising that “the law basically says it just has to be done” (FG3 respondent (R)9). Initiation was viewed as serving the child’s best interest rather than fulfilling a bureaucratic requirement and was considered independent of parental cooperation. Nevertheless, participants described organisational factors that contributed to delayed or missed initiation.

### Organisational conditions

Within CPS, high caseloads and competing demands meant HA were sometimes deprioritised once placement decisions were finalised, with tasks described as “…falling between the cracks…” (FG2R5; FG5R17). Frequent staff changes contributed to inconsistent practices, and established routines were described as guiding everyday work more than the legislative requirement to initiate a HA, “…routines become what we’re used to instead of what the law says…” (FG5R17). Participants also described uncertainty regarding roles and responsibilities between CPS and healthcare that further delayed initiation, particularly when combined with technical information-sharing restrictions for children with protected personal data due to safety concerns and fragmented record systems.

### Professional judgement and conceptions of health needs

Participants expressed strong awareness that children placed in OOHC often have unmet health needs. However, time pressures influenced prioritisation, particularly in temporary placements, and some questioned the value of assessments perceived as superficial or narrowly focused on physical health, “…they are not a top priority and have to wait until we have dealt with the real problems…” (FG4R12). Participants expressed concern that assessments which overlooked mental health needs, trauma exposure and psychological well-being—or were conducted long after placement—might not reflect the child’s current needs.

### Interagency information continuity

Participants reported that previous positive experiences of high-quality HAs facilitated initiation by reinforcing expectations that assessments could provide meaningful information to inform both healthcare and social care planning, as well as CPS assessment of the child’s needs.

Participants described how moves between municipalities or healthcare regions often disrupted continuity of care and fragmented health information, “…before all the records have been obtained…it can take a considerable amount of time to gather everything…” (FG4R11). CPS caseworkers described actively retrieving and compiling medical information from multiple healthcare services across regions, sometimes leading to substantial delays (FG4R13). When comprehensive health records were available, initiation was perceived as more straightforward and justified.

### Scheduling of health assessments

#### Theme 2: negotiation and uncertainty

After initiation, participants described a scheduling phase characterised by interagency negotiation and uncertainty. Coordination with healthcare services, regional variation in routines, access points to healthcare and requirements for parental consent highlighted the absence of a standardised pathway, contributing to uncertainty and delays in the assessment process.

### Consent dilemmas

Participants generally described parents as supportive of HAs; however, obtaining consent remained a potential barrier. In some regions, requirements for multiple parental signatures delayed scheduling by several weeks, “…two and a half weeks before it was cleared…” (FG4R13). Even when children were subject to compulsory care orders, participants emphasised that parental consent was viewed as important for facilitating cooperation, “…it is very good if they consent to a HA, even if it is under LVU (The Care of Young Persons Act) …” (FG2R7).

### Requesting a health assessment

Within their own regions, some participants reported access to clear checklists, templates and designated contact points. Outside their home region, however, procedures were often unclear, “…you just have to rely on what you can find…Google PDFs…” (FG2R5). Dental assessments were frequently described as operating through separate and less transparent processes, “…when it comes to dental care, it is still the case that caseworkers themselves have to find out…” (FG1R2), adding further complexity to coordination.

### Coordination across systems

Coordination during scheduling was described as highly person-dependent. Where established relationships with healthcare professionals existed, communication and scheduling were seen as more straightforward, “…if you have a good relationship with someone, it’s easy…” (FG3R10). In the absence of such relationships, CPS caseworkers relied on individual initiative, informal contacts and local knowledge to navigate the process (FG5R14).

### Routines and communication

Outdated or unclear routines contributed to uncertainty about whether CPS, foster carers or parents should receive appointment notification letters, “…sometimes they call me first, sometimes the foster home…” (FG2; FG4; FG5). Participants expressed a desire for national routines to reduce regional variability and promote more equitable access. Conversely, participants in regions with established routines and templates described scheduling as more predictable, less time-consuming and better coordinated.

### Conduct of the health assessment

#### Theme 3: what we request is not always what we receive

Participants described substantial variation in the content and quality of HAs. While some assessments were experienced as comprehensive and child-centred, others were described as brief examinations focused on growth, cardiovascular and respiratory health, with limited attention to mental health, trauma exposure or exposure to violence.

### Preparation and child-centred approaches

Participants emphasised that many children in OOHC have experienced trauma and require careful preparation, “…many kids have extensive problems…and many need trauma treatment…” (FG2R5). Single-visit assessments were perceived as insufficient for addressing complex needs, “…no one can gather all that information in one appointment…” (FG3R9). Participants also stressed the importance of preparing children for the purpose of the assessment, “…the child should know why they’re going…” (FG2R7). Assessments perceived as more trauma-informed were reported to better prepare children and support their engagement during the examination.

### Competence gaps and assessment fidelity

Assessment quality was perceived to vary markedly across clinicians and healthcare regions. Some healthcare professionals were perceived as knowledgeable and well prepared, while others appeared unfamiliar with the legislative purpose of HAs. Several participants reported that healthcare clinicians appeared to confuse different statutory assessment pathways, resulting in assessments that were narrower in scope than intended, “…we requested a HA…but received a medical certificate issued under the LVU instead…” (FG5R15).

### Collaboration during assessment

CPS caseworkers present during assessments were described as facilitating preparation, communication and reporting, “…the caseworker can encourage the child to talk about what has happened and explain why they are there…” (FG3R10). However, participation was constrained by workload, travel distance and parental reactions. Participants reported challenges coordinating care across paediatric, dental, and child and adolescent mental health services and noted that dedicated regional health assessment teams were perceived to contribute to more consistent and coordinated assessments (FG1R3).

### Reporting and follow-up

#### Theme 4: variable reporting and the challenge of closing the loop

CPS caseworkers described substantial variation in the quality and content of reports returned to CPS following HAs. Reports were described as ranging from detailed and actionable to brief and difficult to interpret due to use of medical terminology, influencing their relevance for healthcare and social care planning and follow-up. Participants also described uncertainty regarding responsibility for acting on identified health needs and ensuring appropriate follow-up, particularly when recommendations spanned multiple services and sectors.

### Clarity and responsibility

Reports containing clear recommendations and explicit follow-up responsibilities were highly valued, “…could actually be used in the investigation and follow-ups …” (FG1R3). In contrast, many reports were perceived to lack this clarity, leaving CPS caseworkers uncertain about next steps, “…I’m a layperson regarding the medical content…” (FG5R18). Participants also described uncertainty regarding responsibility for follow-up after the assessment, noting that expectations were often implicit rather than formally agreed. In particular, participants emphasised that referrals for identified health needs should be initiated by healthcare rather than CPS, “…that is not something we can do…” (FG1R2).

### Using information for care planning

Participants described challenges when reports returned to CPS stated that the health assessment had identified no signs of physical abuse despite concerns about violence or abuse that had contributed to the placement decision. Such findings were perceived as potentially undermining CPS care planning, “…no signs of physical abuse…” (FG2R7). Participants also described dental documentation as particularly fragmented, “…CD ROMs, x rays, handwritten notes…” (FG3R8), making information difficult to access and integrate into planning and follow-up.

### Communicating outcomes to children and carers

Participants expressed uncertainty about how HA results should be communicated to children, parents and foster carers. Narrative summaries that supported children’s understanding of their health history were described as helpful, “…created a sense of coherence…” (FG3R10). CPS caseworkers also described uncertainty regarding responsibility for communicating assessment findings and arranging for follow-up, with these tasks frequently perceived as falling to CPS, contributing to continued coordination challenges (FG3R9; FG5R17).

## Discussion

This study examined how HAs for children in OOHC are initiated, organised, conducted and followed up in routine practice, from the perspective of CPS caseworkers. As illustrated in figure 1, the findings indicate that implementation challenges occur across all phases of the HA pathway and are not primarily driven by lack of awareness of children’s health needs or commitment to ensuring that children receive HAs. Instead, implementation of HAs is shaped by how the statutory requirement is operationalised within organisational and interagency contexts. Participants consistently framed HAs as a child-rights obligation and an important component of safeguarding and care planning, rather than as an administrative requirement, suggesting that implementation challenges occurred despite strong normative commitment among CPS caseworkers. Across the pathway, CPS caseworkers frequently assumed a de facto coordinating role, compensating for fragmented routines, unclear ownership, and limited integration between CPS and healthcare systems. Implementation of HAs emerged as a multidimensional process shaped by structural, ethical and relational factors.

**Figure 1:**
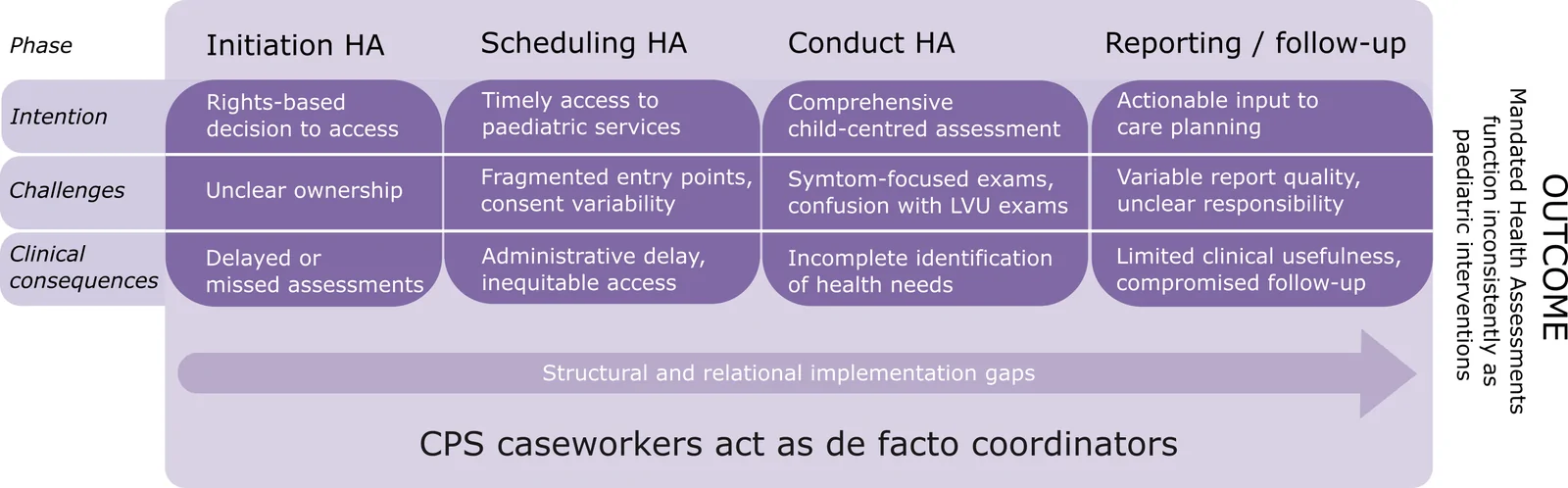
Conceptual overview of implementation challenges across the four phases of mandated health assessments for children in out-of-home care as experienced by child protection services caseworkers. HA, health assessment; LVU (The Care of Young Persons Act) exams: physician assessment as part of compulsory care proceedings; see online supplemental file 1 for more explanation.

These findings align with previous research on inter-organisational collaboration and implementation in child protection and healthcare, which highlights how unclear responsibilities, fragmented communication pathways and weak cross-sector governance undermine coordinated action and service delivery.^28^ They are also consistent with findings from Norway by Haune *et al* (2025), who found that even when comprehensive multidisciplinary HAs identified substantial unmet needs and generated clear recommendations, many children in OOHC did not receive the specialised services recommended.^29^ The authors interpreted this as evidence of a persistent implementation gap between identified needs, professional recommendations and actual service provision, highlighting that children often remained underserved despite their needs being informally recognised and documented.^29^ Our findings suggest that implementation challenges may emerge even earlier in the pathway: before recommendations can be acted on, the assessment and reporting processes need to function effectively in routine clinical practice.

The findings also resonate with ecological models of decision-making and implementation, which emphasise the influence of organisational, inter-organisational and system-level factors on professional practice.^30 31^ An important distinction from much of the existing literature is that the present study focused on a legally mandated intervention. Despite clear legislation, HAs appeared insufficiently institutionalised within routine healthcare practice, suggesting that legal mandates alone do not secure implementation without compatible organisational structures and shared ownership across systems.^23 32^ Participants repeatedly described uncertainty regarding who was responsible for key implementation tasks, including the collection of previous medical records, and highlighted the absence of standardised procedures governing information transfer and follow-up. Although national regulations assign responsibility for collecting relevant health information to healthcare services, the participants nevertheless described practical difficulties in how this responsibility was operationalised. These findings illustrate how responsibility drift can occur at the interface between CPS and healthcare. Similar concerns were identified by Haune *et al* (2025), who questioned who ultimately bears responsibility for meeting the health needs of children in OOHC. They argued that stronger governance mechanisms, clearer allocation of responsibilities and structured pathways for assessment follow-up are needed to ensure that identified needs translate into effective service provision.^29^ Together, our findings and those of Haune *et al* suggest that HAs should not be conceptualised as isolated events but as components of a broader care pathway that includes assessment, referral, coordination and follow-up of identified needs.

While the legal and organisational context is Swedish, the implementation challenges identified mirror barriers described in other CPS and paediatric health systems.^20 22 24 33^

These findings suggest that legislation can create the conditions for implementation but does not ensure coordinated service delivery. This distinction may be relevant internationally, as many mandated child health and child protection interventions rely on effective interagency coordination across organisational and sectoral boundaries.

Participants’ accounts suggest that the clinical and practical value of HA is shaped not only by individual professional commitment but also by how responsibility, competence and information flow are organised. Where assessments were conducted by experienced clinicians supported by clearer routines or trauma-informed approaches, CPS caseworkers perceived them as more meaningful and child-centred. However, reliance on such enabling conditions shifted coordination work onto CPS and reinforced variability rather than consistency. In practice, these implementation gaps risk delaying identification of health needs and interrupting continuity of care for children in OOHC. From a clinical perspective, uncertainty regarding the purpose and scope of HA—particularly in relation to mental health, trauma and exposure to violence—limited clinical fidelity. From an organisational and policy perspective, the findings suggest that clearer shared ownership between CPS and healthcare, better role clarity and more structured reporting and follow-up may be necessary to ensure that mandated assessments contribute to care planning and children’s participation.^34 35^

Several questions remain for future research. Studies incorporating the perspectives of children, carers and healthcare professionals are needed to examine how HA are understood, enacted and experienced across systems. Further research should explore co-produced approaches to the design, delivery and evaluation of HAs, including meaningful involvement of children and young people with lived experience of OOHC. It should also evaluate implementation-focused strategies, such as dedicated assessment teams, single-entry systems or shared care pathways, to determine how organisational integration can be enhanced and reliance on individual coordination reduced. Comparative and longitudinal studies could examine how different organisational models influence the quality, equity and clinical impact of HA over time, informing policy development beyond legislative regulation alone. Finally, research should assess whether improvements in implementation and organisational integration translate into better identification of unmet health needs, improved continuity of care and improved health-related outcomes for children in OOHC.

### Clinical implications for healthcare practice

From a clinical perspective, the findings and participants’ discussions provide insight into how HAs for children in OOHC may become more useful for CPS and care planning. Participants described the most valuable assessments as those conducted by clinicians who were familiar with the statutory purpose of the HA, reviewed relevant health records and adopted a trauma-informed and child-centred approach. CPS caseworkers particularly valued assessments that integrated physical, mental and oral health needs, considered the impact of previous trauma and violence, and resulted in clear, understandable and actionable recommendations. Reports that explicitly outlined recommended actions and follow-up responsibilities were perceived as especially useful for care planning. Participants also emphasised the importance of healthcare services initiating referrals for identified health needs and supporting continuity of care. Together, these findings suggest that the value of a health assessment may depend not only on identifying unmet health needs but also on communicating findings in ways that support coordinated decision-making, care planning and follow-up across healthcare and CPS.

### Strengths and limitations

This study draws on qualitative data from CPS caseworkers across multiple regions within a shared legal framework, allowing for comparison of experiences related to the implementation of HAs under the same statutory mandate. Focusing on CPS is a key strength, as caseworkers are responsible for initiating HAs and play a central coordinating role across the assessment pathway. The consistency of experiences reported across regions and focus groups supports the credibility of the findings.

Several limitations should be acknowledged. The study reflects CPS perspectives only and does not include the views of children, carers or healthcare professionals, which may have provided additional insight into interagency dynamics and implementation challenges. Findings should therefore not be interpreted as direct observations of healthcare practice. The transferability of the findings may also be influenced by variation in the experience and expertise of social workers in Sweden, as well as differences in how municipalities organise their child welfare services (see online supplemental file 1 for background on Swedish CPS). These organisational characteristics may limit transferability to child protection systems organised differently. As a qualitative study, the findings are not intended to be statistically generalisable. Recruitment through professional networks may also have introduced selection bias. Nevertheless, the study provides in-depth insight into implementation processes shaping access to mandated HAs and may offer transferable lessons for similar interagency child health and child protection systems.

## Conclusions

From the perspective of CPS caseworkers, HAs for children in OOHC were perceived to be inconsistently implemented due to structural and interagency barriers rather than lack of professional commitment. Participants described unclear ownership, fragmented routines and limited integration between CPS and healthcare services as factors that reduced the perceived clinical usefulness of assessments and their incorporation into healthcare and social care planning. These challenges may delay identification of health needs and disrupt continuity of care. CPS caseworkers frequently assume a de facto coordinating role to compensate for these systemic shortcomings. Clarifying the purpose and scope of assessments, strengthening professional competence and shared understanding across agencies, and improving the communication and actionability of assessment findings and follow-up recommendations may support more consistent implementation and enhance the contribution of HAs to care planning and follow-up.

## Supplementary material

10.1136/bmjpo-2026-005108 online supplemental file 1

10.1136/bmjpo-2026-005108 online supplemental file 2

10.1136/bmjpo-2026-005108 online supplemental file 3

## Data Availability

Data are available upon reasonable request.
