# Supplementary material for: Structural and interagency influences on mandated health assessments for children in out-of-home care in Sweden: a qualitative study of child protection services workers
Source: BMJ Paediatr Open. 2026 Sep 25;10(1):e005108. doi: 10.1136/bmjpo-2026-005108 (PMC13630047; doi:10.1136/bmjpo-2026-005108)
Supplement: Supplementary file 1 [file bmjpo-2026-005108.reviewer_comments.pdf]

PEER REVIEW HISTORY

BMJ Paediatrics Open publishes all reviews undertaken for accepted manuscripts. Reviewers are asked to complete a checklist review form and are provided with free text boxes to elaborate on their assessment. These free text comments are reproduced below.

ARTICLE DETAILS

**TITLE (PROVISIONAL)**	Structural and interagency influences on mandated health assessments for children in out-of-home care in Sweden: a qualitative study of child protection services workers
**AUTHORS**	Van Agthoven, Godfried<br>Otterman, Gabriel<br>Knez, Rajna<br>Makoroff, Kathi<br>Korhonen, Laura

VERSION 1 – REVIEW

**REVIEWER NAME**	Hjern, Anders
**REVIEWER AFFILIATION**	Centre for Health Equity Studies
**REVIEWER CONFLICT OF INTEREST**	No Conflict of Interest
**AI DISCLOSURE**	No, I have not used AI
**DATE REVIEW RETURNED**	23-Jul-2026

**GENERAL COMMENTS**	This is a most welcome and important contribution to the literature on the health of children in state care. It is well-written and the qualitative analysis used is sound and relevant for the purpose of the study. The authors are well qualified for the task of making sense of the qualitive data collected with their extensive clinical experience from the field.<br>I have but a few comments to facilitate the reading of this important study by the international pediatric readership of BMJ Paediatrics Open.<br>1. For a non-Swedish reader, I think some contextual information about the Swedish organisation of CPS would be helpful. In contrast to many other high income countries like the UK or Norway, the Swedish CPS is located within the general social services in each of Sweden's 290 municipalities. Furthermore, there is no established specialist education for social workers in Sweden, and thus much of the work in CPS is performed by young and inexperienced social workers with limited theoretical knowledge. In the smaller municipalities, CPS is just one of many different tasks for a social worker.<br>2. You have chosen to publish this study in a pediatric journal, but I think more could be done to increase the value for the clinician. The article includes very useful information for the clinician about how to make a high quality health examination in the eyes of the CPS, and particularly the pitfalls of poor ones. I think the core of this information and its relevance for clinical practice could be packaged in a short section in the discussion before the conclusions.<br>3. In a qualitative study you always have to be careful with the language you use in your conclusions. After all, the generalisability of interviews of 18 social workers should be kept in mind. I think the text in article is generally OK in this sense, while the language used

	conclusions in the abstract is questionable. In my opinion, you need a second step in the research process, with a more quantitative approach, to be able to conclude that something is "critical" or "essential". I think that staying with the language in the article, that your findings "suggest" , that gaps "may delay" etc is more scientifically appropriate.<br>4. The article assumes that health examinations alone are useful to improve the health of children in state care. This may seem like common sense, but there really isn't much evidence in literature to support that assumption. A recent Norwegian study (see Haune et al https://doi.org/10.1177/25161032251324861) did not show much relation between recommendations from the health examination and the actual health care use during the following year. Maybe you want to add something about this in the discussion related to your finding that health examination should include initiation and maybe also follow-up of the needs identified?<br>5. I was a little surprised that the interviewed stated that it was problematic for them to collect the health records needed for the health examination. This is a practice that was changed in the new guidelines of 2020 from the National Board of Health and Welfare (HSLF-FS 2019:19). According to these guidelines (5 och 10 §§) it is the health care organisation that has the responsibility to collect this information. Whether this has been implemented in every region in Sweden, I don't know, but in the Stockholm region that's certainly how we do it.

VERSION 1 – AUTHOR RESPONSE

Reviewer 1

#1 This is a most welcome and important contribution to the literature on the health of children in state care. It is well-written and the qualitative analysis used is sound and relevant for the purpose of the study. The authors are well qualified for the task of making sense of the qualitive data collected with their extensive clinical experience from the field.

Response: We thank the reviewer for these encouraging comments and for recognising the potential contribution of our study.

#2 For a non-Swedish reader, I think some contextual information about the Swedish organisation of CPS would be helpful. In contrast to many other high income countries like the UK or Norway, the Swedish CPS is located within the general social services in each of Sweden's 290 municipalities. Furthermore, there is no established specialist education for social workers in Sweden, and thus much of the work in CPS is performed by young and inexperienced social workers with limited theoretical knowledge. In the smaller municipalities, CPS is just one of many different tasks for a social worker.

Response: Many thanks for this valuable suggestion. To provide greater context for international readers, we have expanded Supplementary File 1 with additional information on the organisation of the Swedish child protection system, including its municipal structure, the integration of child protection services within the broader social services system, and variation in local organisation and workforce composition across municipalities. We have also added background information on Sweden’s statutory framework for health assessments of children in out-of-home care, LVU medical examinations, and the referral pathways within the healthcare system. In addition, we added a sentence in the Strengths and Limitations section of the manuscript directing readers to Supplementary File 1 and noting that variation in the organisation and experience of the child welfare

workforce may influence the transferability of the findings.

#3 You have chosen to publish this study in a pediatric journal, but I think more could be done to increase the value for the clinician. The article includes very useful information for the clinician about how to make a high-quality health examination in the eyes of the CPS, and particularly the pitfalls of poor ones. I think the core of this information and its relevance for clinical practice could be packaged in a short section in the discussion before the conclusions.

Response: Thank you for this valuable comment. We agree that the findings have important implications for paediatric clinicians. In response, we have added a dedicated section entitled Clinical implications for paediatric practice immediately before the conclusions. The broader implications for research and policy remain addressed separately in the Discussion.

#4 In a qualitative study you always have to be careful with the language you use in your conclusions. After all, the generalisability of interviews of 18 social workers should be kept in mind. I think the text in article is generally OK in this sense, while the language used conclusions in the abstract is questionable. In my opinion, you need a second step in the research process, with a more quantitative approach, to be able to conclude that something is "critical" or "essential". I think that staying with the language in the article, that your findings "suggest" , that gaps "may delay" etc is more scientifically appropriate.

Response: Thank you for this important comment. We agree that the conclusions of a qualitative study should be expressed cautiously and avoid implying levels of certainty that are not supported by the study design. While the wording in the manuscript was generally consistent with this approach, the conclusions in the abstract were more definitive than intended. We have therefore revised and tempered the wording in the abstract conclusions and key messages, using language that better reflects the qualitative nature of the study and the exploratory character of the findings.

#5 The article assumes that health examinations alone are useful to improve the health of children in state care. This may seem like common sense, but there really isn't much evidence in literature to support that assumption. A recent Norwegian study (see Haune et al https://doi.org/10.1177/25161032251324861) did not show much relation between recommendations from the health examination and the actual health care use during the following year. Maybe you want to add something about this in the discussion related to your finding that health examination should include initiation and maybe also follow-up of the needs identified?

Response: Many thanks for this valuable comment. We have expanded the Discussion by incorporating findings from Haune et al.

#6 I was a little surprised that the interviewed stated that it was problematic for them to collect the health records needed for the health examination. This is a practice that was changed in the new guidelines of 2020 from the National Board of Health and Welfare (HSLF-FS 2019:19). According to these guidelines (5 och 10 §§) it is the health care organisation that has the responsibility to collect this information. Whether this has been implemented in every region in Sweden, I don't know, but in the Stockholm region that's certainly how we do it.

Response: Thank you for this important clarification. We agree with the reviewers comment. However, participants in our study, speaking from a CPS perspective, nevertheless described challenges related to obtaining and compiling health information from different healthcare providers and regions. We have therefore added a discussion point highlighting the distinction between formal regulatory requirements and their implementation in routine practice.
