# Supplementary material for: Structural and interagency influences on mandated health assessments for children in out-of-home care in Sweden: a qualitative study of child protection services workers
Source: BMJ Paediatr Open. 2026 Sep 25;10(1):e005108. doi: 10.1136/bmjpo-2026-005108 (PMC13630047; doi:10.1136/bmjpo-2026-005108)
Supplement: Supplementary file 2 [file bmjpo-10-1-s001.docx]

**Supplementary File 1.**

*Background on Sweden’s Statutory Framework for Health Assessments of Children in Out-of-Home Care*

Sweden’s Act (2017:209) on Health Assessments for Children and Adolescents in Out-of-Home Care was introduced to ensure that children placed in foster care or residential care receive comprehensive health assessments. Under the Act, Sweden’s 21 independent health care regions are required to carry out these assessments following a referral from Child Protection Services (CPS), with the costs borne by the health care sector. The country’s 290 autonomous municipalities, which administer CPS, are responsible for identifying children in need of assessment and referring them to appropriate health care providers.

The mandated assessment covers physical, mental, and oral health, reflecting the well-documented risk of unmet health needs among children in out-of-home care. Its purpose is to identify and address both immediate and longer-term health concerns, thereby supporting improved health outcomes for this particularly vulnerable group.

To support implementation of the Act, the National Board of Health and Welfare (NBHW, Socialstyrelsen), which oversees both the social welfare (CPS) and health care systems, issued binding regulations and policy guidance in 2019 (NBHW, 2019). These documents provide direction to health care professionals and CPS caseworkers by clarifying responsibilities and coordinating procedures for conducting the assessments.

Despite the legislation and accompanying regulatory framework, national reports indicate that fewer than half of children in out-of-home care undergo the mandated health assessments.

*Referral System in Swedish healthcare*

In Sweden, access to paediatric care is generally referral-based. General practitioners typically provide primary care for children and refer cases requiring specialised paediatric expertise to paediatricians. Paediatricians mainly deliver secondary and tertiary care, managing more complex or specialised health conditions.

*LVU medical examination versus health assessment*

A LVU^[1]^ medical examination resulting in a medical certificate (Section 32, The Care of Young Persons (Special Provisions) Act; SFS^[2]^ 1990:52) is typically a focused physician assessment commissioned by social services to support evidentiary decision-making in compulsory care proceedings. By contrast, the statutory health assessment for children and adolescents entering out-of-home care under Act (2017:209) is a structured, rights-based clinical assessment delivered by regional health services to identify physical, mental, and oral health needs and to initiate follow-up care at the start of placement. Although both processes may occur in the same case, they serve distinct legal and clinical purposes.

*Child Protection Services / Child welfare services in Sweden*

Child welfare in Sweden is integrated within the general municipal public social services in each of the 290 municipalities. These services are broadly comparable to what is termed CPS in some other countries but are integrated within the wider municipal social services system. I Sweden CPS rely on family service models and place the family at the centre, rather than focusing primarily on the child. Caseworkers may also have broader social work responsibilities, especially in smaller municipalities, with child protection and out-of-home care comprising only part of their overall caseload. Most out-of-home placements are made voluntary under the Social Services Act, while compulsory placements are governed by the LVU. CPS rely heavily on voluntary interventions and act as a social safety net, with coercive interventions under the LVU legislation applied only when voluntary measures have been insufficient or exhausted. CPS caseworkers do not unilaterally decide on coercive measures, as the Social Services Board within the municipality must petition an independent Administrative Court, which determines whether the legal conditions for compulsory care are met. Workforce experience and staffing may vary across municipalities, with smaller municipalities sometimes having more limited access to experienced caseworkers tasked with planning child welfare interventions, including out-of-home placements ^iii^.

1. LVU: Lagen med särskilda bestämmelser om vård av unga = The Care of Young Persons Act
2. SFS: Svensk författningssamling = Swedish Code of Statutes

   ^iii^ Shanks, E. (2023). No choice? Hiring agency social workers in the Swedish personal social services. European Journal of Social Work, 26(5), 896–907. https://doi.org/10.1080/13691457.2022.2147147
