# Supplementary material for: Structural and interagency influences on mandated health assessments for children in out-of-home care in Sweden: a qualitative study of child protection services workers
Source: BMJ Paediatr Open. 2026 Sep 25;10(1):e005108. doi: 10.1136/bmjpo-2026-005108 (PMC13630047; doi:10.1136/bmjpo-2026-005108)
Supplement: Supplementary file 3 [file bmjpo-10-1-s002.docx]

**Supplementary File 2**.

Interview guide for the semi-structured focus group interviews.

*Total 60-90 minutes*

Opening Question (group cohesion purpose) 5-10 minutes:

- *Tell us about your experience with placing children in social care.*

Key Questions (questions/answers most important for analysis) 50-70 minutes:

- *What is your general conception on health assessments of children placed in out-of-home care?*
- *What is the attitude and child perspective of child protection services (CPS) case workers regarding the health of children in out-of-home care?*
- *What are the barriers and hinder CPS case workers experience in the process of initiating health assessments?*
- *What is the (collective) experience of CPS case workers regarding the communication and collaboration with health care workers during and after health assessments?*
- *How is quality assured during the whole process of initiation, assessment, report, and collaboration?*

Closing Question (reflection, additions to earlier discussions) 5-10 minutes:

- *What do you consider the most important when it comes to health assessments?*
- *What was not mentioned in the focus group today which you think is important to reflect on?*
