# Supplementary material for: Structural and interagency influences on mandated health assessments for children in out-of-home care in Sweden: a qualitative study of child protection services workers
Source: BMJ Paediatr Open. 2026 Sep 25;10(1):e005108. doi: 10.1136/bmjpo-2026-005108 (PMC13630047; doi:10.1136/bmjpo-2026-005108)
Supplement: Supplementary file 4 [file bmjpo-10-1-s003.docx]

**Supplementary File 3.
Consolidated criteria for Reporting Qualitative research (COREQ) Checklist**

| **Item** | **Guide question** | **Guide question answer** | **Reported on page No.** |
| --- | --- | --- | --- |
| 1. Interviewer / facilitator | Which author(s) conducted the interviews or focus groups? | The first author (GvA) moderated and conducted all focus group discussions. | 5 |
| 2. Credentials | What were the researcher’s credentials? | MSc, MD and PhD candidate | 1 (title page/ ORCID) |
| 3. Occupation | What was the researcher’s occupation at the time of the study? | Consultant paediatrician and doctoral researcher. | 1 (title page/ ORCID) |
| 4. Gender | Was the researcher male or female? | Male. | 5 |
| 5. Experience and training | What experience or training did the researcher have? | The moderator had prior experience conducting qualitative interviews and professional collaboration with child protection services (CPS) staff. | 5 |
| 6. Relationship established | Was a relationship established prior to study commencement? | No prior personal relationships with participants were established before recruitment. | 15 |
| 7. Participant knowledge of the interviewer | What did participants know about the researcher? | Participants were informed about the researcher’s professional background as a paediatrician and the purpose of the study. | 5 |
| 8. Interviewer characteristics | What characteristics were reported about the interviewer? | Reflexive practice was used to acknowledge the potential influence of the researcher’s professional background on data collection and interpretation. | 6 |
| 9. Methodological orientation and theory | What methodological orientation underpinned the study? | Reflexive thematic analysis with inductive coding, guided by decision-making ecology. The Consolidated Framework for Implementation Research was used interpretively in the discussion. | 6 & 14 |
| 10. Sampling | How were participants selected? | Purposive snowball sampling of CPS caseworkers with experience in out-of-home care placements. | 5 |
| 11. Method of approach | How were participants approached? | Recruitment occurred via professional networks, national events, Barnafrid outreach, webpage announcements, and contact with social services managers. | 5 |
| 12. Sample size | How many participants were in the study? | Eighteen CPS caseworkers participated. | 5 & 6 |
| 13. Non‑participation | How many declined or dropped out? | Thirty-four CPS caseworkers expressed interest in participating; ultimately, 18 were allocated to five focus groups within the project timeframe. | 5 |
| 14. Setting of data collection | Where was the data collected? | Data were collected through secure, encrypted Zoom® video focus groups. | 5 |
| 15. Presence of non‑participants | Was anyone else present? | At least one additional author was present as a silent observer during the focus groups. | 6 |
| 16. Description of sample | What were the key characteristics of participants? | Participants represented rural, urban, and city municipalities across Sweden; variation in years of professional experience was reported. | 6 |
| 17. Interview guide | Were questions provided to participants? | A semi‑structured interview guide was used (Supplementary File 2), piloted and refined prior to data collection. | 5 |
| 18. Repeat interviews | Were repeat interviews carried out? | No repeat interviews were conducted. | 6 |
| 19. Audio/visual recording | Were interviews recorded? | Focus group discussions were video‑recorded. | 5 |
| 20. Field notes | Were field notes made? | Observations and reflections were recorded immediately after focus groups. | 6 |
| 21. Duration | What was the duration of interviews? | Focus groups lasted approximately 90–105 minutes. | 5 |
| 22. Data saturation | Was data saturation discussed? | Meaning saturation was assessed during data collection and used to determine when no further focus groups were required. | 5 |
| 23. Transcripts returned | Were transcripts returned to participants? | Transcripts were not returned to participants for comment or correction. | 6 |
| 24. Number of data coders | How many coders were involved? | All coding was performed by the authors GVA, RK and LK. Coding and interpretations were refined through iterative discussions within the research team. | 6 |
| 25. Description of coding tree | Was a coding framework described? | Codes were developed inductively and iteratively; themes were refined through team discussions. | 6 |
| 26. Derivation of themes | Were themes identified in advance or derived from data? | Themes were derived inductively from the data using the decision-making ecology framework lens. | 6 |
| 27. Software | What software was used? | NVivo® qualitative data analysis software. | 6 |
| 28. Participant checking | Did participants provide feedback on findings? | Participants did not provide feedback on the findings. | 6 |
| 29. Quotations presented | Were participant quotations presented? | Illustrative quotations were included in the Results section, identified by participant codes. | 7-12 |
| 30. Data and findings consistent | Was there consistency between data and findings? | Findings were grounded in participant quotations and closely linked to the analytic themes. | 14 |
| 31. Clarity of major themes | Were major themes clearly presented? | Four major themes were presented, structured across the phases of the health assessment process. | 6-7 |
| 32. Clarity of minor themes | Were minor themes or subthemes described? | Subthemes were clearly identified within each phase and major theme. | 7-12 |
